# The Effect of Family Empowerment Model on Quality of Life in Children with Epilepsy in South of Iran, 2018: A Randomized Controlled Clinical Trial

**DOI:** 10.22037/ijcn.v15i4.30119

**Published:** 2021

**Authors:** Hamid NEMATI, Zahra MAHDAVI KHANOUKI, Mohammad GHASEMPOUR, Ahmad Ali AMIRIFAR, Fatemeh ALAEI KARAHROUDI, Maryam GHOLAMI

**Affiliations:** 1 Shiraz Neuroscience Research Center, Shiraz University of Medical Sciences, Shiraz, Iran; 2 Instructor, Department of Nursing and Midwifery, Islamic Azad University, Kerman Branch, Kerman, Iran; 3 Medical Library and Information Sciences, Central Library, School of Medicine, Kerman University of Medical Sciences, Kerman, Iran.; 4 Faculty of Nursing and Midwifery, Shahid Beheshti University of Medical Sciences, Tehran, Iran; 5 Nursing Expert, Shiraz University of Medical Sciences, Shiraz, Iran

**Keywords:** Child, Epilepsy, Family, Empowerment, Quality of Life, Iran

## Abstract

**Objectives:**

Epilepsy is a chronic disease of the nervous system, which remarkably affects children’s performance and behaviors. Epileptic children are at greater risk of cognitive and behavioral disorders compared to healthy children. In this regard, a variety of factors associated with this disease may affect the patients' families.

**Materials & Methods:**

The present study was a randomized controlled clinical trial, which aimed to evaluate the effect of family empowerment on the quality of life in epileptic children referred to the concerned centers (the Bessat Clinic affiliated to the Kerman University of Medical Sciences and Shiraz’s Imam Reza Clinic). The participants, who were 80 parents with epileptic children meeting the inclusion criteria, were assigned into two experimental and control groups. In this study, the Quality of Life in Childhood Epilepsy Questionnaire (QOLCE) was used to collect the required data from the parents.

**Results:**

The studied children’s age ranged from 4-8 years. These results revealed a statistically significant difference between the two groups regarding the level of changes in all dimensions, indicating that the experimental group’s mean score of quality of life in different dimensions increased (*P* <0.05 in all dimensions).

**Conclusion:**

The implementation of the family-centered care plan by care providers, based on patient-family support relationships, the detection of their strengths and weaknesses, prioritization of the provided services, and effective interaction with the health team would increase the family and staff’s satisfaction, reduce the costs, and improve the outcome of the disease.

## Introduction

Epilepsy is a chronic disease of the nervous system, which remarkably affects children’s performance and behaviors. Epileptic children are at greater risk of behavioral and cognitive impairment than healthy children (1). The International League Against Epilepsy (ILAE) accepted recommendations of a task force altering the practical deﬁnition for particular circumstances that do not meet the two unprovoked seizures criteria. The task force proposed that epilepsy is a brain disease with (a) at least two unprovoked (or reﬂex) seizures occurring more than 24 h apart, (b) one unprovoked (or reﬂex) seizure and a probability of further seizures similar to the general recurrence risk (at least 60%) after two unprovoked seizures occurring over the next 10 years, or (c) the diagnosis of an epilepsy syndrome (2). About 0.5-1 percent of children are suffering from epilepsy, 20% of whom are resistant to treatment (3). Studies have indicated that 4-10% of children have experienced at least one case of seizure before the age of 16 years, with a majority of them being below three years old. Almost 150000 children experience asymptomatic seizures annually, 20% of whom also experience epilepsy (4). According to the World Health Organization (WHO), a seizure is a disease affecting about 65-100 persons per 100000 people. Out of 26 persons, one is directly affected by seizure disorders, and one-tenth of the individuals experience such disorders during the first three years of their lives. The prevalence of the disorders may be associated with decreased child growth, lifelong disability, and dependency, as well as increased economic costs (5). According to the Guideline Committee of the American Epilepsy Society, 50000-150000 Americans experience epilepsy annually. The disease leads to death in <a3% of children (6). Epilepsy is one of the most severe neurological disorders, requiring long-term treatment due to its chronic nature (7). 

Epilepsy is a chronic disease affecting children's performance and behaviors. Epileptic children are at greater risk of cognitive and behavioral disorders than healthy children (1). A variety of factors associated with this disease may affect the patients' families. The families’ routine daily activities, which were to meet the needs of the family members, may shift to activities meeting the needs of the child suffering from the disease (8). Nowadays, considering the patient's family is one of the most critical components of patient care since the family is in charge of providing care for the patient and plays a crucial role in accelerating the patient’s recovery process. Family is an integrated system, and one family member can threaten the whole system, thereby arousing fear, weakness, loss of hope, and physical and mental exhaustion in other family members (9). Family is considered a crucial component of child care program during the disease. In this regard, the family members' involvement plays a significant role in child care since children are directly dependent on family members in taking self-care. Parents' involvement in the care procedure of a hospitalized child can be a challenging experience for the parents and the members of the medical team (10). Accordingly, family-centered care is an innovative approach to planning, implementing, and evaluating health care. It is underpinned by mutually-beneficial cooperation among patients, families, and health care providers. The four main components of family-centered care are respect, information reception, involvement in care, and collaboration (11).

The concept ‘family-centered approach’ was first used in the 1950s when Carl Rogers introduced the client-centered approach. In a client-centered approach, the clients, not service providers, manage and control the disease. In the same decade, Beret and Scherz introduced the family-centered service to provide services to children and their families (12). Training parents and their involvement in the care procedure during the hospitalization, especially at discharge time, is of paramount importance in this approach. Previous studies have indicated that developing a treatment plan and answering questions is one of the preliminary needs of such parents. Training is one of the principles in promoting community health. In this regard, in early 1973, the American Nursing Association recognized patient and family training as a critical responsibility in the realm of ​​nursing duties (13). There is a correlation between diseases and quality of life, and physical disorders and physical symptoms directly affect all aspects of quality of life (14). The primary goal of treatment, especially in chronic diseases, is to promote the quality of life by decreasing the side-effects of the disease, implying that patients with chronic diseases should not experience a lower quality of life (15). As seizure and epilepsy disorders interfere with motor and learning activities, they pose some problems in school activities and leisure time of the patient; hence, special attention is needed to be paid to promoting the quality of life in these children (16). A shift in the disease treatment from traditional approaches to empowering patients and families and promoting their involvement in the care process indicate that the focus is now on health, prevention, and health training, not exclusively on disease and treatment (17). In this regard, empowerment would also result in positive self-esteem, goal-achieving ability, a sense of control over life, and hope for the future (18).

## Materials & Methods

The present study was a randomized controlled clinical trial, which aimed to evaluate the effect of family empowerment on the quality of life of epileptic children referred to the concerned centers. According to the medical records of patients referred to Bessat Clinic affiliated to the Kerman University of Medical Sciences and Shiraz’ Imam Reza Clinic during the last six months and given the prevalence of epilepsy reported in some studies (4, 5), 80 parents with epileptic children, who met the inclusion criteria, were selected and studied. After obtaining the informed consent, they were randomly assigned into the experimental (n=40) and control (n=40) groups. The research inclusion criteria were as follows:

1. Having epileptic children aged between 4-8 years, whose disease had been diagnosed at least six months ago and had already started the treatment and required at least one year of follow-up, based on their medical records and physician’s diagnosis;

2. Providing informed consent and being willing to participate in research;

3. Holding high school diploma or master’s degree and having adequate monthly income;

4. Not participating in similar training programs;

5. Suffering from no physical and mental disease disrupting daily activities; and

6. Not being a healthcare staff.

The research exclusion criteria included:

1. Parents’ unwillingness to cooperate and non-referrals of the child at due times;

2. Parents’ non-participation in training sessions; and

3-Hospitalized children diagnosed with fever–induced seizure.

Sampling was performed randomly with permuted blocks. Accordingly, a number was randomly selected, and then binary blocks were selected as AB (0-0) and BA (5-9) with 40 consecutive numbers. Group A was considered as the experimental group, and Group B was the control group. The convenient sampling method was used as long as the required number of samples was achieved. Then the samples were randomly assigned to two experimental and control groups. The study sample encompassed the parents of epileptic children. Their informed consent was also obtained to participate in the present study. In this study, the Quality of Life in Childhood Epilepsy Questionnaire (QOLCE) was used to collect the required data. The questionnaire assesses the quality of life of epileptic children and is derived from the health-related quality of life (HRQoL) assessment tool. In this study, the parents were asked to complete the questionnaire. HRQoL is used to assess the health care and medical interventions required in the clinical and social domains (19) as children with seizure disorders are at increased risk of behavioral and emotional problems, family and social decline, and a poor quality of life (20). QOLCE assesses children's function in four subscales: cognitive function (22 items), motional factors (17 items), social function (7 items), and physical function (9 items). Each item is scored on a five-point Likert scale (very low = 0, low = a mean score of 25, moderate= a mean score of 50, acceptable = a mean score of 75, and high = a mean score of 100). The validity of the QOLCE Questionnaire was assessed by Goodwin et al. (2015), and they reported its validity at a high level. 

The intervention and empowerment program in the experimental group were performed during two training sessions. In the first session, the parents were trained on the course of the disease, its clinical symptoms and diagnosis, and probable complications by lectures, question and answer techniques, pamphlets. The second session included face-to-face training on the provision of care in seizure, existing treatments, post-treatment recovery, and the need for long-term individual and group follow-ups by educational pamphlets. After one month of training, the parents completed the Quality of Life Questionnaire once more. In the control group, after completing the questionnaire, the educational pamphlet was submitted to the parents to observe the ethical issues. The quantitative data were presented as mean ± SD, and the qualitative data were presented as frequency (%). Independent and paired-sample t-tests were used for statistical analysis, and Mann-Whitney U test would be used if the data were not normally distributed. Data were analyzed by SPSS software version 16, and *p*= 0.05 was set as the level of significance.

## Results

**Figure 1:**
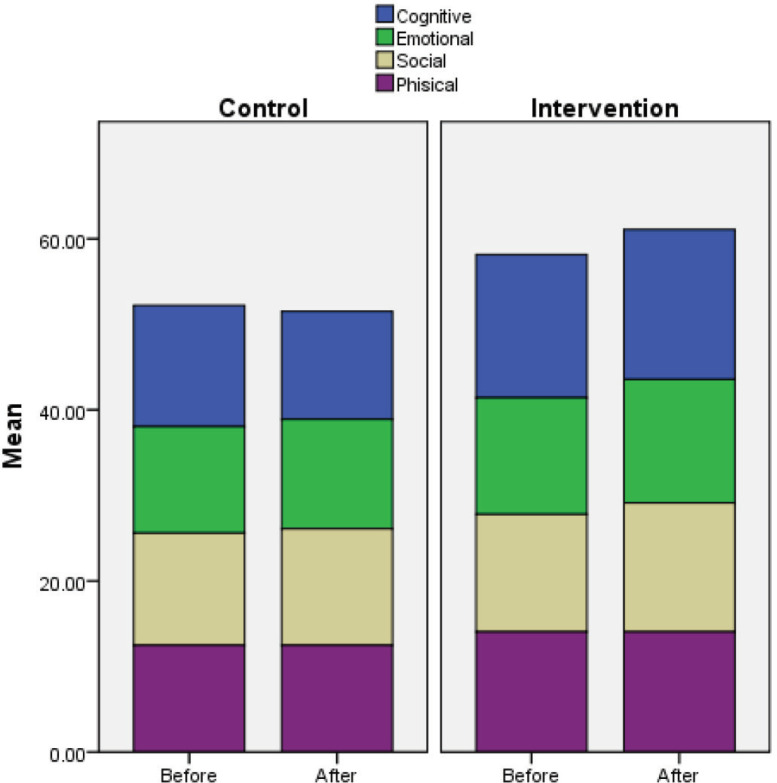
Total score of Quality of life before and after intervention in experimental and control groups

In this study, 80 participants were divided into two experimental (n=40) and control (n=40) groups, among whom there were 43 (53.8%) males. The studied children’s age ranged from 4-8 years, with the mean age of 5.85 ± 1.53 years. Table 1 shows the demographic characteristics of the two groups. According to the results, there was no significant difference between the two groups in terms of their demographic features (*P* <0.05). In other words, the two groups were homogeneous in this regard. Table 2 compares the different dimensions of quality of life in the epileptic children and presents the changes in the mean scores of quality of life regarding different dimensions. Accordingly, there was a statistically significant difference between the two groups regarding the changes in all dimensions, so that the mean score of quality of life in all dimensions increased in the experimental group, compared to the control group (*P* <0.05). The inter-group analysis also revealed that the increased values in the experimental group were statistically significant (*P* <0.05 in all dimensions). However, in the control group, the changes in emotional, social, and physical dimensions were not significant (*P* <0.05), and only the cognitive function revealed a significant change (a decrease in cognitive function score). The overall score of quality of life also showed a significant difference between the pre-test and post-test scores (*P* <0.001). Moreover, in the intra-group analysis, the differences between pre-test and post-test scores were significant in the experimental group (*P* <0.001); however, no significant difference was not observed in the control group in this regard (*P* = 0.850) (Table 2). The results indicated that the intervention could increase the quality of life score in the four dimensions (namely cognitive function, emotional function, social function, and physical function) in the experimental group. However, in the control group, the quality of life in these dimensions decreased, and no significant increase was noticed. Figure 1 illustrates the findings for the total quality of life scores.

**Table1:** Participants’ demographic information

*p*-value	Control (n=40)	experimental (n=40)	Variables	
0.116	(5/62)25	(45)19	Gender (male)	
0.246	57/1±05/6	51/1±65/5	Age	
0.570	(5/17)7	(15)6	below diploma	Education
	(15)6	(5/37)15	Diploma	
	(5/67)27	(5/47)19	Academic	
0.437	(35)14	(5/22)9	Self-employed	Occupation
	(5/32)13	(35)14	Employed	
	(5/32)13	(5/42)17	Housewife	
0.130	(90)36	(5/77)31	Satisfactory	Level of income
	(10)4	(5/22)9	Non-Satisfactory	
0.370	(5/52)21	(5/42)17	Father	Patients’ family member
	(5/47)19	(5/57)23	Mother	
0.796	(45)18	(5/42)17	1	Number of children in family
	(45)18	(5/42)17	2	
	(10)4	(15)6	3	
0.179	(60)24	(45)18	Family history	

**Table 2:** Comparing the quality of life score between experimental and control groups

*p*-value	Control	Study	Variables			
0.002	32/18±64.56	89.9±04.67	Before			Cognitive
<0.001	12/7±45/50	50 /9±05/75	After			
<0.001	07/18±19/6-	66/2±01/3	Mean(sd)	Change		
	(0-7/22-)27/2-	(41/3-13/1)84/2	Med(Q1-Q3)			
	0.036	<0.001	p-value			
0.018	48/8±81/49	59/8±44/54	Before			Emotional
<0.001	98/6±13/51	79/7±83/57	After			
0.029	84/8±33/1	08/4±38/3	Mean(sd)	change		
	(0/4-47/1-)73/0	(88/5-47/1)14/2	Med(Q1-Q3)			
	0.350	<0.001	p-value			
0.438	59/16±5/52	68/11±0/55	Before			Social
0.004	40/8±55/54	86/8±26/60	After			
0.002	12/18±05/2	49/7±26/4	Mean(sd)		change	
	(67/2-14/7-)0	(1/7-0)5/3	Med(Q1-Q3)			
	0.476	<0.001	p-value			
0.071	85/16±41/45	65/9±04/51	Before			Physical
<0.001	83/7±86/49	04/7±18/56	After			
0.008	17/19±44/4	91/5±13/5	Mean(sd)		change	
	(5/5-7/2-)0	(3/8-0)5/5	Med(Q1-Q3)			
	0.151	<0.001	p-value			
0.010	07/12±09/51	83/6±88/56	Before			Total
<0.001	22/5±50/51	56/5±08/61	After			
<0.001	55/13±40/0	01/3±20/4	Mean(sd)		change	
	(8/0-18/8-)11/1-	(65/5-43/1)91/3	Med(Q1-Q3)			
	0.850	<0.001	p-value			

Data are presented as mean ± sd and med (Q1-Q3).

## Discussion

In their study, Novriska et al. (2014) investigated the behavioral problems in children with seizure disorders and reported that about 19% of children with seizure disorders had behavioral problems affecting their quality of life. They concluded that behavioral screening disorders and timely diagnosis and treatment are required to eliminate such side-effects (21). Megan Murray (2015) in a study entitled ‘family-centered care for children with a head injury’ revealed that recognizing the family structures and needs, using effective communication strategies, and identifying cultural barriers to the provision of family-centered care would improve the overall quality of life in the children (22). Nohi et al. implemented a family-centered care program for children with gastrointestinal infections and revealed that the implementation of this care program, compared to the conventional method, aroused lower anxiety in the parents (23). Minuei et al. evaluated the effect of family empowerment on the quality of life of children with chronic kidney failure, and they found out that empowerment interventions and family-centered training reduced costs and promoted the quality of life in such children (24). Furthermore, in their study entitled “The Effect of Self-Management Empowering Model on Spiritual Health of Mothers of Children with Cerebral Palsy,” Zare et al. showed that empowerment programs implemented for such mothers could improve their spiritual health and the overall quality of life in these mothers and their families (25). These findings are consistent with those of the present study. Dalvand et al. also evaluated the effectiveness of the family-centered approach by reviewing relevant articles and documents in Iranian and non-Iranian databases during 1985-2012. They reported that the family-centered approach was effective for children, parents, families, and service providers and promoted the quality of service provision process and the families’ satisfaction. Family-centered care has been less implemented in Iran due to the lack of sufficient knowledge and education on family-based services, lack of applied educational materials, and a foundation for medical treatment and rehabilitation in accordance with a medical model (12). The previous studies have indicated that providing continuous training and empowering families influence the proper function of follow-up care. Hence, it is necessary to provide accurate information for families by regular and continuous training. Nowadays, the provision of training for patients and families is considered a part of health staff's vital activity. Researchers believe that family empowerment promotes accountability, interaction with the health team, better responses to treatment while decreasing medical costs, length of hospital stay, and disease complications.

**In Conclusion , **In general, family-centered care is a care philosophy highlighting the central role of the family in children's lives. It is also an essential component of child care, maintaining the integrity of the child's family, providing unique care, and enhancing child health. The implementation of the family-centered care plan by care providers, based on patient-family support relationships, the detection of their strengths and weaknesses, prioritization of the provided services, and effective interaction with the health team would increase the family and staff’s satisfaction, reduce the costs, and improve the outcome of the disease. The family empowerment program was developed to highlight the effectiveness of the family role in motivational, psychological (namely self-confidence, self-control, and self-efficacy), knowledge, attitude, and perceived threat dimensions. Many experts believe that empowerment is a dynamic, interactive, and social process, which is shaped during communication with others, improves the quality of life in individuals with chronic diseases, accountability, and interaction with health authorities, increases satisfaction and better response to treatment, decreases the frequency of complications and medical costs, and nurtures a positive view to the disease. Accordingly, patient and family empowerment has been of great significance in nursing and medical studies as such it should be considered as an essential part of the nursing profession. The main goal of the family-centered empowerment program is to empower the family system (namely patients and other family members) to promote health.
